# In vitro- and in vivo-produced male dairy calves show molecular differences in the hepatic and muscular energy regulation^†^

**DOI:** 10.1093/biolre/ioac131

**Published:** 2022-06-29

**Authors:** María B Rabaglino, Jan Bojsen-Møller Secher, Poul Hyttel, Haja N Kadarmideen

**Affiliations:** Quantitative Genetics, Bioinformatics and Computational Biology Group, Department of Applied Mathematics and Computer Science, Technical University of Denmark, Kgs. Lyngby, Denmark; Department of Veterinary Clinical Sciences, University of Copenhagen, Frederiksberg C, Denmark; Department of Veterinary Clinical Sciences, University of Copenhagen, Frederiksberg C, Denmark; Quantitative Genetics, Bioinformatics and Computational Biology Group, Department of Applied Mathematics and Computer Science, Technical University of Denmark, Kgs. Lyngby, Denmark

**Keywords:** bioinformatics, system biology, multi-omics, fetal programming

## Abstract

In cattle, the in vitro production (IVP) of embryos is becoming more relevant than embryos produced in vivo, i.e. after multiple ovulation and embryo transfer (MOET). However, the effects of IVP on the developmental programming of specific organs in the postnatal calves are yet unknown. Previously, we reported an epigenomic and transcriptomic profile of the hypothalamus–pituitary–testicular axis compatible with its earlier activation in IVP calves compared to MOET animals. Here, we studied the hepatic and muscular epigenome and transcriptome of those same male dairy calves (*n* = 4 per group). Tissue samples from liver and semitendinosus muscle were obtained at 3 months of age, and the extracted gDNA and RNA were sequenced through whole-genome bisulfite sequencing and RNA-sequencing, respectively. Next, bioinformatic analyses determined differentially methylated cytosines or differentially expressed genes [false discovery rate (FDR) < 0.05] for each Omic dataset; and nonparametrically combined genes (NPCG) for both integrated omics (*P* < 0.05). KEGG pathways enrichment analysis showed that NPCG upregulated in the liver and the muscle of the IVP calves were involved in oxidative phosphorylation and the tricarboxylic acid cycle. In contrast, ribosome and translation were upregulated in the liver but downregulated in the muscle of the IVP calves compared to the MOET calves (FDR < 0.05). A model considering the effect of the methylation levels and the group on the expression of all the genes involved in these pathways confirmed these findings. In conclusion, the multiomics data integration approach indicated an altered hepatic and muscular energy regulation in phenotypically normal IVP calves compared to MOET calves.

## Teaser

Transcriptomic and epigenomic results suggest that aerobic respiration was upregulated in both liver and muscle, while protein synthesis was increased in the liver but downregulated in the muscle of in vitro produced calves compared to in vivo counterparts.

## Graphical Abstract

**Graphical Abstract:**
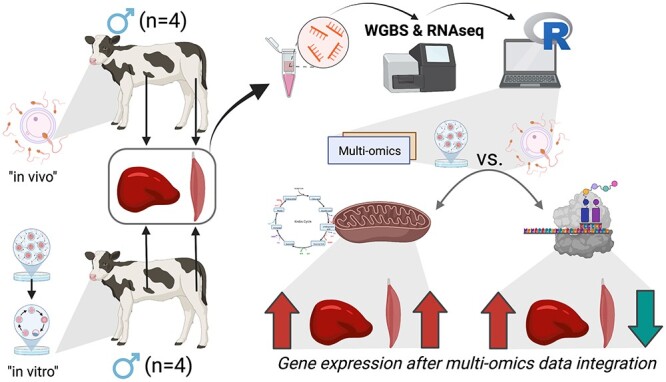

## Introduction

The application of assisted reproductive technologies (ART) in cattle has been reported since the 1900s, with the successful use of artificial insemination (AI) (reviewed in Ref. [1]). However, ART involving embryo manipulation is relatively more recent. The transfer of in vivo produced embryos was achieved in the early 1950s [2], while in vitro fertilization and the birth of the in vitro produced (IVP) calves were gradually developed and refined over the 1980s and 1990s [3, 4]. Today, the use of these technologies, i.e. IVP of bovine embryos and multiple ovulation and embryo transfer (MOET), are extensively used worldwide to improve genetic gains in beef and dairy cattle (reviewed in Refs. [5, 6]). The use of MOET was for many years more extensively used than IVP. However, this trend has been reversed during the last few years [7], given the lower costs and improvements in the techniques employed for IVP. In this regard, enhancement of the culture media has reduced the incidence of the most common unwanted phenotype in IVP calves: large offspring syndrome (LOS) (reviewed in [8]) although this phenomenon is not completely eliminated yet. LOS is an overgrowth syndrome caused mainly by alterations in the epigenetic profile in various organs, both in imprinted and nonimprinted regions, which are associated with changes in gene expression [9–12]. On the other hand, IVP calves born normal did not differ in their reproductive functions and lactation performance from calves generated by AI [13, 14]. However, changes at the molecular level in the organs of the apparently normal IVP calves are still unexplored.

In a previous article, we demonstrated that healthy dairy male IVP calves, of 3 months age, presented an epigenomic/transcriptomic profile compatible with early activation of the hypothalamus–pituitary–gonadal (HPG) axis when compared to the MOET calves [15]. There were no differences regarding body weight at birth and growth rate among these animals. Our results suggested a premature maturation of the IVP calves, which could have impacted the age at puberty, although this is unknown so far. In the present study, we complete this previous report by analyzing the epigenomic and transcriptomic data from muscle and liver samples obtained from the same IVP and MOET calves at 3 months of age. Both organs play a crucial role in the programming of metabolism regulation during fetal life (reviewed in Ref. [16]). Thus, we hypothesize that the molecular profile of these organs is compatible with altered metabolism in IVP calves when compared to the MOET animals, even when they were phenotypically similar. The objective of this study was to apply integrative multiomics and bioinformatics approaches to explore the muscular and hepatic epigenomic and transcriptomic differences between phenotypically normal IVP and MOET male calves.

## Materials and methods

### Animals

The donors, recipients, and calves were all housed in the same nucleus herd, ensuring minimal differences in the housing environment. The Danish Animal Experiments Inspectorate characterized the IVP, superovulation, and embryo transfer procedures as part of the nucleus herd breeding program Tirsvad Holstein. Therefore, we did not need a license for these practices. The euthanasia procedures were approved by the local ethical and administrative committee at the Department of Veterinary Clinical Sciences at the University of Copenhagen (license number 2020-006). Table 1 describes the parental combination used to produce the ovum pick-up (OPU)-IVP or MOET embryos.

**Table 1:** Donors and sires employed to produce the calves for this study. CKR is the unit number for animal registration in the Danish Central Register of Livestock. MOET: embryos were produced in vivo (ovarian superovulation followed by embryo collection and transfer). IVP: embryos were produced in vitro (ovarian mild stimulation, OPU, and cultured/fertilized in a serum-free media)

Animal IDs	Groups	Mother CKR	Recipient CKR	Father
5833	IVP	24680-05142	24680-05115	My Dream P RC
5841	IVP	24680-05142	24680-05139	My Dream P RC
3208	IVP	24680-05142	40156-02786	Builder P
3209	IVP	24680-05142	42042-03225	Builder P
5932	MOET	24680-04912	24680-05227	Solitær P
3213	MOET	24680-04912	49578-03835	Simon P
3217	MOET	24680-05142	53871-04210	BuilderP
3218	MOET	24680-05142	40156-02795	BuilderP

### Production of the OPU-IVP embryos

OPU was performed after a mild 2-day stimulation protocol using 75 IU of follicle-stimulating hormone (FSH) and luteinizing hormone (LH), once a day each day (Pluset, Scanvet, Denmark) starting 9–11 days after detection of spontaneous estrous (mid-luteal phase), 3 days before OPU. Further, 24 h after the last FSH/LH injection, and just before OPU, the donor was given epidural anesthesia (Lidocain). An ECM scanner (ECM France) with a 5 MHz transducer was used to visualize the donor’s ovaries. Follicles were aspirated transvaginally using a 17-gauge needle connected to a Minitube GmbH aspiration pump. The oocytes were collected in OPU media (IVF-Bioscience, UK) and scored according to the layers of cumulus cells, color, and homogeneousness of ooplasma.

Chemically defined media from IVF Bioscience was used for oocyte search, in vitro maturation, in vitro fertilization, and in vitro culture. All steps were performed according to the manufacturer’s guidelines, as previously described [17]. The embryos were transferred fresh 7–8 days after fertilization.

### Production of the multiple ovulation and embryo transfer embryos

Beginning in the mid-luteal phase, 6–13 days after registered estrus, the donors were superstimulated with a total of 800 IU FSH and 800 IU LH (16 ml Pluset, Scanvet, Denmark) over 4 days in a decreasing dose schedule, according to the manufacturer’s guidelines. Briefly, twice a day (08:00 and 20:00), 3 ml or 150 IU (day 1), 2.5 ml or 125 IU (day 2), 1.5 ml or 75 IU (day 3), and 1.0 ml or 50 IU (day 4) of FSH and LH were injected intramuscular. On the fourth day of stimulation, luteolysis was induced with 0.5 mg cloprostenol (Estrumate, MSD Animal Health, Denmark). AI was performed 2 and 3 days after luteolysis was induced. Embryo flushing was performed 7 and 8 days after insemination, and embryos were transferred fresh. The recipients were synchronized with 0.5 ml cloprostenol given 24 h before the donors were given cloprostenol. The recipients received epidural anesthesia (Lidocain) before the transfer, and a single embryo was transferred ipsilateral to the corpus luteum.

### Calves

The calves were born at Tirsvad Holstein (Denmark) and housed there until 2.5 months of age. All animals in this study were born at term from February 2020 to June 2020, and they were all Holstein males (*n* = 4 per group). Birth weight did not differ between IVP and MOET calves (38.25 ± 2.62 kg vs. 36.25 ± 2.75 kg, respectively, *P* > 0.05). Calves in both groups were raised in similar conditions until euthanasia, performed at around 3 months of age (101.75 ± 2.5 vs. 102.67 ± 1.15 days for IVP and MOET calves, respectively), except for one MOET calf that was 144 days old. Bodyweight gain per day from birth to euthanasia was similar among calves in both groups (0.88 ± 0.1 kg vs. 0.83 ± 0.1 kg for IVP and MOET calves, respectively, *P* > 0.05). For euthanasia, the calves were transported to the Large Animal Hospital of UCPH (Taarstrup, Denmark). They were housed for 14 days in the hospital stables and euthanized with 100 mg/kg of pentobarbital sodium (Dechra Veterinary Products A/S, Denmark). Tissue samples from several organs were obtained in less than 10 min after euthanasia and immediately snap-frozen in liquid nitrogen. All samples were biobanked in liquid nitrogen. For the current study, samples from the semitendinosus muscle in the left leg, and from the right lobe of the liver were employed.

### gDNA/RNA extraction, library preparation, and sequencing

Tissue samples were shipped in dry ice to the BGI TECH SOLUTIONS Company (Hong Kong), which performed the gDNA and RNA extraction, quality control, library preparation, and sequencing. All samples met the requirements for library preparation [guanine and cytosine (G and C) content between 35% and 65% for extracted gDNA; adequate concentration and quantity, purity as OD260/280 = ~1.8 – 2.0 and RIN > 8 for the extracted RNA]. The gDNA samples were subjected to whole-genome bisulfite sequencing (WGBS), through bisulfite library preparation and PE100 sequencing with 45Gb clean data per sample on DNBSEQ. RNA-sequencing (RNA-seq) was run in the RNA samples through nonstranded and polyA-selected mRNA library preparation and PE100 sequencing with 5 Gb clean data per sample on DNBSEQ. For both techniques, after library preparation and sequencing, raw data with adapter sequences or low-quality sequences were filtered to remove contamination and obtain valid data. This step was completed by the SOAPnuke software developed by the BGI Company. Clean raw data were generated in the FASQ format.

### Bioinformatic analysis

**Figure 1:**
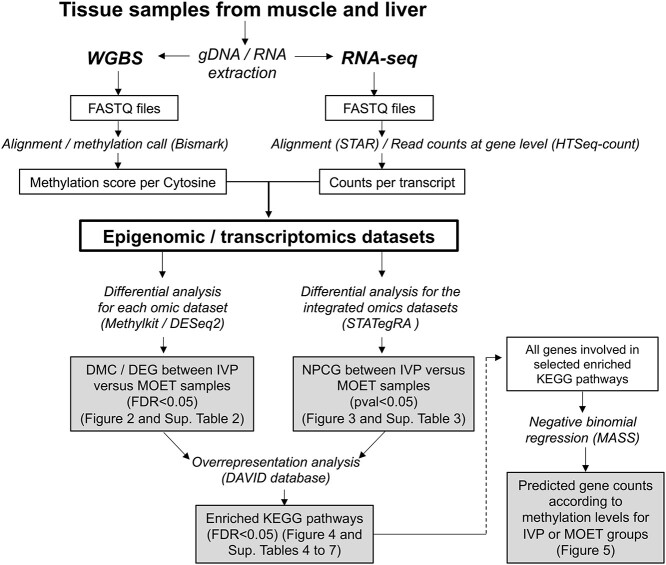
Bioinformatic pipeline and main outputs of the study. The scheme represents the workflow employed in the samples from the liver and muscle organs obtained from 3 months old IVP or MOET male calves. The methods are detailed in italics (the software used are in parenthesis), while the outputs are shown in the squares. Shaded squares denote the main results from the study (corresponding tables or figures are indicated in parenthesis). The dashed arrow means that the information from a given result was used for the other result. MOET: embryos were produced in vivo (ovarian superovulation followed by embryo collection and transfer). IVP: embryos were produced in vitro (ovarian mild stimulation, OPU and cultured/fertilized in a serum-free media). DMC: Differentially methylated cytosines. DEG: differentially expressed genes. NPGC: nonparametrically combined genes.

The bioinformatic pipeline and main outputs of this study are shown in Figure 1. The WGBS clean reads were aligned to the bovine reference genome (bosTau 9) using the Bismark Bisulifte Read Marker (v 0.22.3) [18]. All covered cytosines were used for calculation of global CpG methylation level in Bismark using the following formula: percent global methylation = (number of methylated cytosines/total number of cytosines) × 100. The RNA-seq read pairs were mapped to the bovine reference genome (bosTau 9) with STAR aligner (v. 2.7) [19], generating the genome index with the gene *Bos taurus* release 102 annotations. Read counts were estimated at the gene level using HTSeq-count (v. 0.11.1) [20]. Samples’ distribution and clustering, according to the methylated CpG or gene expression, were assessed through principal component analysis (PCA) and hierarchical clustering, respectively, using internal packages of R.

Data are deposited in The National Center for Biotechnology Information’s Gene Expression Omnibus (GEO) and are accessible through the GEO accession number GSE176219.

### Determination of differentially methylated cytosines and differentially expressed genes

The binary alignment map files generated from the Bismark software after processing the WGBS files were analyzed using the Bioconductor package methylKit [21] for the R software. Read coverages lower than 10 counts or higher than the 99.9th percentile were filtered out to discard low coverage and clonal reads. The effect of age was controlled by removing the component associated with this variable. Identification of differentially methylated cytosines (DMC) between IVP and MOET groups was performed by logistic regression, where the “group” variable was used to predict the log-odds ratio of methylation proportions. The logistic regression model was fitted per methylated region, testing if the group vector had any effect on the outcome variable or not. *P*-values were adjusted to *q*-values using the sliding linear model method [22], and DMC were defined as those with *q* < 0.05. Annotation of DMC was performed with the Bioconductor/R package Genomation [23]. Browser extensible data files of CpG islands and RefSeq database for the bosTau9 assembly were downloaded from the UCSC table browser (https://genome.ucsc.edu/cgi-bin/hgTables). All DMC were first annotated with the nearest (no specific cut-off) transcription start site (TSS). Next, DMC were annotated with gene structures (promoter, exon, intron, CpG islands, or shores). Promoters and CpG shores were defined as ±1000 bp and ± 2000 bp of the TSS and CpG islands, respectively.

The read count files (text files) obtained after processing the RNA-seq files were employed to determine the differentially expressed genes (DEG) with the DESeq2 package [24] for the R software. The effect of age was controlled with the sva package [25]. Next, the gene expression counts were normalized by library size with DESeq2 methods. The differential analysis was performed by fitting a logistic regression model to the gene counts, modeled by a negative binomial distribution, and p-values were adjusted with the Benjamini–Hochberg method. The Wald test statistic was employed to test for model significance. The DEG between IVP and MOET groups were defined as those with false discovery rate (FDR) <0.05.

Given that the sample size was low, we performed a power analysis a posteriori with the ssizeRNA_vary function of the ssizeRNA package for R [26]. This function calculates the power for different sample sizes in two-groups RNA-seq experiments with variable means and dispersions among genes. The calculation was performed by specifying an alpha (FDR) of 0.05, the corresponding proportions of non-DEG, the mean counts in the MOET group, and the dispersion.

### Integration of both omics datasets

Identification of relevant genes when combining both omics data for each tissue was done through a nonparametric combination (NPC) methodology [27], applied with the omicsNPC function [28] of the STATegRA package [29]. Briefly, each dataset was analyzed separately first, to compute *P*-values when contrasting both groups. Next, these *P*-values were combined through the Fisher function, using 1000 permutations. Genes that were significant (*P*-value <0.05) after the combination were defined as “nonparametrically combined genes” (NPCG). In other words, these muscular or hepatic genes are associated with the embryo origin of the calves when considering both transcriptomic and epigenomic data. Identified NPCG were further evaluated according to their mRNA expression in IVP or MOET samples, through hierarchical clustering and heat map. The clustering was made using Spearman Rank Correlation as a similarity metric and centroid linkage as a clustering method, implemented with the Cluster 3.0 software [30]. The resulting dendrogram and the heat map were visualized with Java TreeView [31]. Genes in the resulting clusters were compared to the DEG, and to the overlapping DEG and genes associated with the DMC, through Venn Diagrams, using Venny 2.1 (https://bioinfogp.cnb.csic.es/tools/venny/).

### Functional analysis

The EntrezID corresponding to the annotated TSS related to DMC in a genic region, to the DEG, or to the NPCG, was interrogated for enriched KEGG pathways using the Database for Annotation, Visualization and Integrated Discovery (DAVID; [32]), using the Functional Annotation Clustering tool. Only clusters with functional terms enriched at FDR < 0.05 were retained.

Based on these results, some pathways were analyzed to determine if the methylation levels and the group (IVP or MOET) were influencing the expression of the genes involved in such pathways. For this, the effect of methylation levels, the group, and the interaction among these variables, on the read counts for each gene was modeled through a negative binomial regression using the MASS package for the R software [33]. Each term was considered significant at *P* < 0.05. If the interaction was not significant, it was dropped from the model. The complete list of genes’ Entrez IDs belonging to the selected pathways was downloaded from the KEGG pathway database (https://www.genome.jp/kegg/pathway.html). Next, for each pathway and organ, the methylation proportions for each gene in each sample were obtained across the whole genome (annotated CpG with nearest TSS). If more than two regions included the same Entrez ID, the methylation proportions were averaged. A similar step was performed for the transcriptome data if more than two Ensembl IDs were encoding for the same Entrez ID, although the average counts were rounded. In addition, some of these pathways were graphed with the Pathview package [34]. Briefly, this tool set maps the data with the pathway of interest and renders the pathway graph. Genes in these pathways with more expression in IVP or MOET groups were colored in red or green, respectively.

## Results

### Methylation and expression data mapping

Data generated from the WGBS method had on average, a 67.4% of unique mapping rate to the *B. taurus* genome, ranging from 65.1% in the liver to 69.8% in muscle. Per group, the averages were 68.1% and 66.57% for IVP and MOET groups, respectively. From these mapped reads, 72.1% (67.8–72.1%) of the cytosines belonged to CpG, and only 1.1% to CHG or CHH (which were not considered in further analyses). Transcriptomic data generated from the RNA-seq method resulted in a high proportion of reads with unique alignment to the *B. taurus* genome: 96.3% on average (95.5–96.7%). Further, 80.9% (76.5–84.6%) of these reads were assigned to Ensembl IDs (78.9% in liver and 82.9% in muscle), and around 80.6% in both groups.

Table S1 contains the details about the results from the mapping and methylation call steps for the epigenomic data and mapping and read counting steps for the transcriptomic data. Sample relatedness according to overall CpG methylation levels or expression levels is shown in Figures S1 and S2, respectively.

### Identification of DMC and DEG in liver and muscle of IVP versus MOET

**Figure 2:**
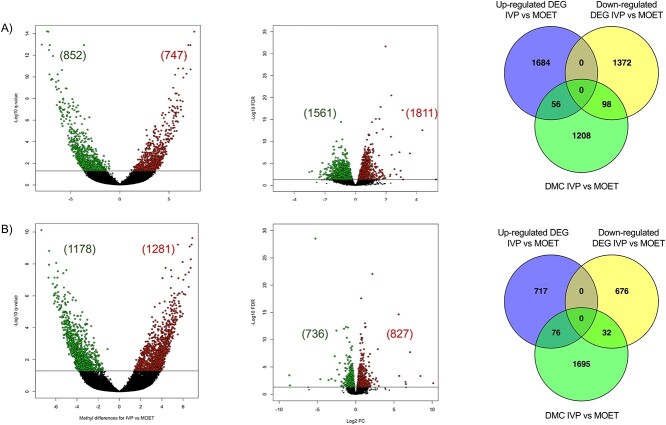
Differentially methylated cytosines (DMC) and differentially expressed genes (DEG) in the liver (A) or muscle (B) of IVP versus MOET calves. Volcano plots in the left represent the number of hyper (red dots) or hypo (green dots) DMC; while the ones in the center depict the upregulated (red dots) or downregulated (green dots) DEG. Venn Diagrams in the right show the overlap between up- and down-regulated DEG and genes associated to DMC; 154 for liver and 108 for muscle. IVP: embryos were produced in vitro (ovarian mild stimulation, OPU and cultured/fertilized in a serum-free media). MOET: embryos were produced in vivo (ovarian superovulation followed by embryo collection and transfer).

The number of DMC and DEG, respectively, were 1599 and 3372 for the liver; and 2459 and 1563 for the muscle (FDR < 0.05). There were 154 and 108 overlapping genes between DEG and genes associated with the DMC for liver and muscle, respectively (Figure 2 and Table S2). For the DEG, the power for a sample size of *n* = 4 was around 80% and 72%, considering that 12.2% and 5.7% of the 27 607 transcripts were DEG for liver and muscle, respectively.

### Identification of NPCG between IVP and MOET calves

**Figure 3:**
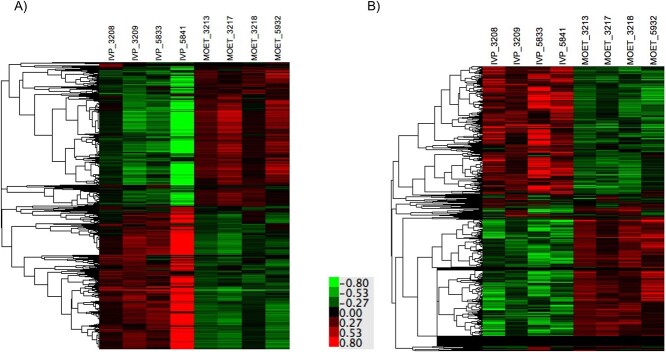
Hierarchical clustering and heat map for the mRNA expression of the nonparametrically combined genes (NPCG) in liver (A) and muscle (B). These genes have a *P*-value < 0.05 after application of the nonparametric combination method, which considers the comparison between IVP and MOET calves for both epigenomic and transcriptomic datasets. IVP: embryos were produced in vitro (ovarian mild stimulation, OPU, and cultured/fertilized in a serum-free media). MOET: embryos were produced in vivo (ovarian superovulation followed by embryo collection and transfer).

**Figure 4:**
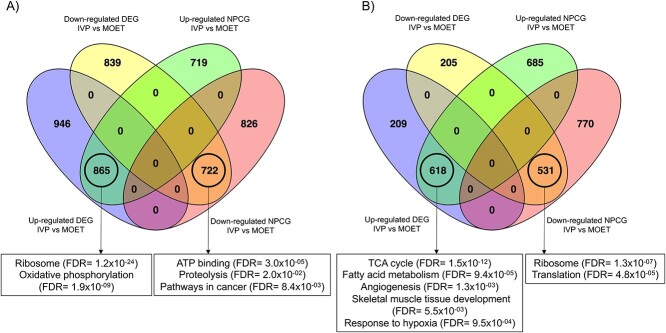
Venn diagrams comparing differentially expressed genes (DEG) and clusters of nonparametrically combined genes (NPCG) in liver (A) and muscle (B). DEG are up- or down-regulated in IVP samples relative to MOET samples. NPCG are those with a *P*-value of <0.05 after application of the nonparametric combination method, which considers the comparison between IVP and MOET calves for both epigenomic and transcriptomic datasets. The clusters were determined as shown in Figure 2. Functional terms displayed in the boxes were enriched by the overlapping genes. IVP: embryos were produced in vitro (ovarian mild stimulation, OPU, and cultured/fertilized in a serum-free media). MOET: embryos were produced in vivo (ovarian superovulation followed by embryo collection and transfer).

Application of the NPCG methodology to the individual *P*-values for genes associated with methylated cytosines, and expressed genes, when IVP calves were compared to the MOET counterparts, resulted in 3606 and 2733 NPCG for liver and muscle, respectively, for which the combined *P*-value was <0.05 (Table S3). Figure 3 shows the mRNA expression of these NPCG in the liver or muscle of IVP and MOET calves. In the liver, there were two main clusters with 1548 and 1584 NPCG upregulated in the MOET and IVP calves, respectively. In the muscle, the two main clusters contained 1303 NPCG more expressed in the IVP calves and 1301 NPCG upregulated in MOET calves. Several NPCG were identified as DEG, following the same direction for the expression levels in IVP or MOET samples. For example, in the liver, around half of the NPCG on each cluster were overlapping with around half of the up or downregulated DEG. For muscle, around 44% of the NPCG overlapped with about 73% of the DEG (Figure 4). Furthermore, 70.1% and 73.1% of the overlapping DEG and genes associated with DMC, for liver and muscle, respectively, were also classified as NPCG (Figure S3).

### Functional analysis of DEG, of genes associated with DMC, and of NPCG

*Liver:* The functional analysis of the upregulated DEG showed that the top clusters contained strongly enriched functional terms related mainly to ribosome and translation, and mitochondria and oxidative phosphorylation. However, the downregulated DEG enriched focal adhesion and extracellular matrix interaction, among other functional terms (Table S4). Genes near hypomethylated cytosines were associated with lipid metabolism/secondary metabolites biosynthesis, transport, catabolism, and ATP binding; but no functional terms were enriched with genes related to hypermethylated cytosines. However, the cluster of NPCG more expressed in the IVP calves was enriched for similar functional terms as the DEG (ribosome, translation, and mitochondrial respiration). NPCG upregulated in the MOET calves were enriched in ATP binding, protein kinase, and cancer-related pathways (Table S5). Matches between enriched functional terms by DEG or by NPCG occurred because of the overlapping genes between them, which were associated with the common functional term (Figure 4A).

**Figure 5:**
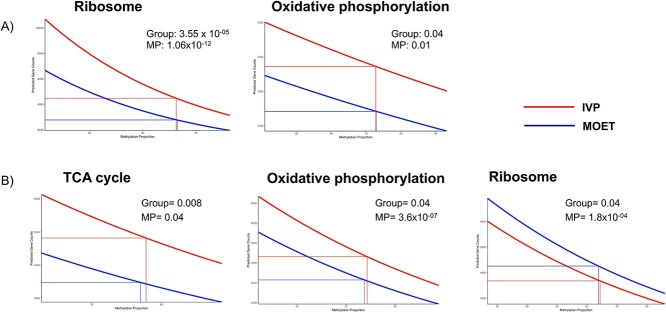
Prediction of read counts for genes involved in specific pathways from 10% to 90% of methylation proportion (MP). The boxes show the *P*-values for the effect of MP or the group (IVP or MOET) in the expression of all genes in each pathway in the liver (A) or (B) muscle of IVP and MOET calves. Vertical and horizontal lines indicate the predicted gene counts for each group at the average MP. IVP: embryos were produced in vitro (ovarian mild stimulation, OPU, and cultured/fertilized in a serum-free media). MOET: embryos were produced in vivo (ovarian superovulation followed by embryo collection and transfer).

The negative binomial model showed that both the methylation levels and the group affected the expression of genes involved in the ribosome and oxidative phosphorylation KEGG pathways. There was a negative correlation between methylation levels and gene expression, which was predicted to be higher in samples from the IVP calves at any methylation level (Figure 5A).

*Muscle*: Upregulated DEG in the muscle of the IVP calves strongly enriched functional terms related to aerobic respiration, the tricarboxylic acid (TCA) cycle, angiogenesis, fatty acid metabolism, skeletal muscle development, and response to hypoxia. Contrary to the liver, downregulated DEG were associated with ribosome, translation, and amino acid biosynthesis (Table S6). Genes near the TSS of hypermethylated cytosines were involved in protein tyrosine phosphatase activity, whereas those related to hypomethylated cytosines only enriched glycosylation. NPCG upregulated in IVP calves were enriched by 12 annotation clusters containing similar functional terms as the upregulated DEG. However, NPCG upregulated in MOET calves were enriched by three main annotation clusters with functional terms related to ribosome and translation (Table S7). As for the results from the liver, overlapping DEG and NPCG were enriched by the common functional terms (Figure 4B).

Methylation proportions and the group affected the expression of genes involved in the TCA cycle, angiogenesis, muscle development, and ribosome, with a negative correlation between methylation levels and gene expression. For genes in all the pathways, except for ribosome, the expressions were predicted to be higher in the IVP group, whereas the inverse occurred for genes in the ribosome pathway (Figure 5B).

## Discussion

Results from the present study demonstrate that the embryo origin influenced the overall hepatic and muscular epigenome and transcriptome of the 3 month calves (Figures S1 and S2). Although the sample size was limited, findings were highly significant and indicated that calves produced through IVP showed aberrant hepatic and muscular epigenomic and transcriptomic profiles that, when compared to MOET calves, were clearly compatible with the following biological imbalances: increased aerobic respiration and thus energy generation in both organs; and notoriously, increased protein synthesis in the liver but decreased in muscle. Indeed, illustrating the expression levels for genes in oxidative phosphorylation and ribosome pathways distinctly highlight the similarities and divergences, respectively, between liver and muscle from IVP and MOET calves (Figure S4). Furthermore, a comparison between up-or downregulated DEG in the liver and muscle of the IVP calves compared to the MOET calves demonstrates that common DEG between upregulated DEG in the liver and downregulated DEG in muscle strongly enriched the ribosome pathway and the translation process. However, common upregulated DEG in both organs of the IVP calves enriched oxidative phosphorylation and fatty acid metabolism (Figure S5). Alterations of these functional terms occurred both at the transcriptomic and epigenomic levels. Although genes near the TSS of DMC did not enrich them, these pathways were associated with the NPCG, i.e., significant genes considering both types of data (Tables S5 and S7). Furthermore, by modeling the influence of the methylation level for all the genes involved in them (regardless of the statistical difference) and the group (IVP or MOET) on their mRNA expression, it became apparent how these terms were affected on muscle and liver of the IVP calves (Figure 5).

These metabolic differences for both organs in IVP versus MOET calves could be a potential consequence of the in vitro process per se. The physicochemical, oxidative, and energetic conditions of the medium used for the in vitro culture of the oocytes and/or embryos have strong effects on embryo developmental programming [35], impacting its epigenome and, in turn, generating consequences that can be retained into postnatal life. Epigenetics refers to “heritable changes in gene expression without altering the DNA sequence” [36], which can be altered by the environment, inducing modifications that remains in long term and even cross generations [37]. Although several investigations have improved the media for the IVP of bovine embryos, it is well accepted that the in vitro process still represents a stressful environment for the embryo, affecting it at the molecular level [38–44]. This effect is comparable to other adverse maternal situations, such as nutritional insults. For example, in a recent report, Chaput and Sirard [45] collected embryos from 60 days postpartum dairy cows presenting high levels of hydroxybutyrate, an indicator of negative energy balance. The transcriptome and epigenome of these embryos exposed to the maternal unfavorable conditions were coincident with previous findings for IVP embryos: the embryos switch to an “economy” mode by activating the mammalian target of rapamycin (mTOR) pathway and tumor protein 53 factor, whereas the mitochondrial function is reduced. Thus, the most affected pathways in these embryos were related to metabolism, including protein synthesis, and oxidative phosphorylation [45]. Accordingly, the response of IVP embryos to the stressful in vitro environment is reflected in mitochondrial dysfunction, not only impacting the energy regulation but also the production of methyl groups associated with one-carbon metabolism controlling histone acetylation and DNA methylation [46], and thus, mediating epigenetic modifications.

In a recently published study, we characterized the epigenetic and transcriptomic modifications of the IVP embryos compared to MOET embryos through the application of a multiomics data integration approach, to identify genes that were temporally differentially expressed and differentially methylated between IVP and MOET embryos from the blastocyst stage to the elongated conceptus [47]. We focused on the changes that could impact the trophectoderm function, which is the outermost layer of the conceptus that is in contact with the endometrium. A meta-analysis of 10 publicly available transcriptomic and epigenomic datasets revealed a cluster of genes with a strong deviation in their expression between IVP and MOET embryos at day 13 when the elongation process is initiated. Several of these genes were significantly related to the focal adhesion pathway. Interestingly though, the oxidation–reduction process and mitochondrial matrix were also among the affected functional terms by genes in this cluster (*P* < 0.05).

**Figure 6:**
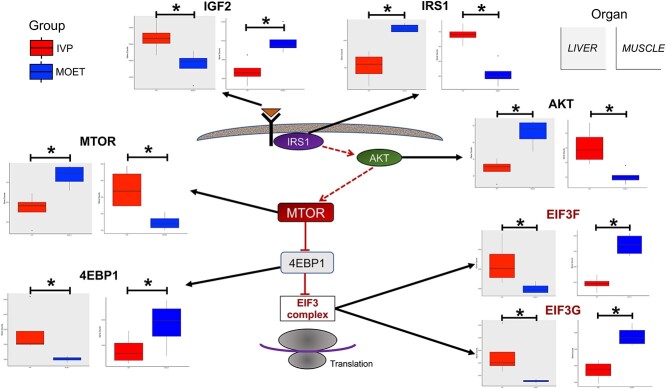
Expression levels for certain genes in the insulin/IGF-AKT–mTOR pathway. Genes followed opposite expression in the liver (gray background) and muscle (white background) of IVP calves (red boxplots) compared to MOET calves (blue boxplots). All the genes in the figure were differentially expressed at false discovery rate < 0.05 (*). Dashed arrows mean an indirect effect of one gene on the other. IVP: embryos were produced in vitro (ovarian mild stimulation, OPU, and cultured/fertilized in a serum-free media). MOET: embryos were produced in vivo (ovarian superovulation followed by embryo collection and transfer).

Therefore, IVP conceptuses might present alterations in metabolic regulatory pathways from the blastocyst stage resulting in impaired trophoblast function, which is responsible for placental development. Indeed, previous reports in cattle have shown that the IVP process can impact the placenta by increasing the diameter and decreasing the thickness of the placentomes [48] and impairing blood vessel development [12, 49–51]. These aberrations might lead to placental insufficiency, a well-established cause of developmental programming, as the fetus might be chronically exposed to low oxygen levels (reviewed in Ref. [52]). Furthermore, the fetus can acquire a thrifty metabolic phenotype, which can even persist after birth [53, 54]. For example, in humans, 12-year-old children born with small size and low birth weight have similar metabolic rates as average-sized children, although energy production is mostly obtained from lipid oxidation rather than glucose oxidation [55]. In sheep, lambs that suffered intrauterine growth restriction (IUGR), a common consequence of placental insufficiency [56], not only are born with lower weight but later in life, they present lower contents of skeletal muscle protein, greater adiposity, and altered glucose metabolism and liver function [57–59]. Thus, metabolic adaptations and substrate utilization in fetal tissues can impact the organs’ functionality permanently.

Of all the organs in the body, the skeletal muscle and the liver are prone to develop persistent metabolic adaptations. Although they account for around 15% of the total weight of the late gestational fetus [60], combined they are responsible for around 50% of the total fetal oxygen consumption, and so they are the largest metabolic fetal organs [61, 62]. All calves in the present study presented comparable body weights at birth and at 3 months of age, so they were not suffering from IUGR or any other apparent maternal insult during fetal life. However, although subtle abnormalities in the fetal–placental unit can be compensated during gestation, the epigenome and transcriptome of these animals can be modified and remain altered during the postnatal life. Evidently, IVP animals presented abnormalities in metabolic pathways in skeletal muscle and liver compared with the MOET animals. Aerobic respiration was stimulated in both organs while notoriously, protein synthesis strongly diverged: it was increased in the liver while decreased in the muscle.

The explanation behind these observations might reside in how these organs react to the main oxidizable substrates used for energy production and tissue growth in the fetus, such as glucose, lactate, and amino acids [63]. The main orchestrators of nutrient partitioning are insulin and the insulin-growth factors (IGF). Briefly, insulin mediates its response through the insulin receptor (IR) and the insulin receptor substrate (IRS) proteins 1 and 2. The downstream signaling includes activation of the phosphoinositide 3-kinase and protein kinase B (AKT) pathways, and the classic p44/p42MAPK (ERK1/2) signaling cascade [64], which in turn, activates mTOR. However, mTOR regulates energy-sensing pathways, coordinating mRNA translation and mitochondrial energy production, to regulate cellular proliferation and growth rates [65, 66]. Indeed, protein synthesis needs to be closely coordinated with the energy yield, as it is one of the most energy-consuming processes in the cell [67, 68].

Interestingly, the mRNA expressions for several of the crucial players in the IGF/insulin-AKT–mTOR pathway were opposite in the liver and the muscle of the IVP calves, and significantly different from the expression in MOET calves (Figure 6 and Table S2). IGF2 is a well-known imprinted gene synthesized mainly by the fetal pancreas [69] and liver [70], which plays an important role in organ growth. In the IVP calves, compared to the MOET animals, IGF2 mRNA expression was upregulated in the liver and downregulated in muscle, while IR and IRS1 were downregulated in the liver and IRS1 upregulated in muscle. Regulation of translation is achieved through activation of mTOR, which phosphorylates and activates the eukaryotic initiation factor 4E binding protein 1 (4EBP1), causing their dissociation from the eukaryotic translation initiation factor 3 (EIF3) complex [71]. Although mTOR was downregulated in the liver and upregulated in muscle, 4EBP1 and all the 12 subunits of EIF3 were upregulated in the liver and inhibited in the muscle of the IVP calves compared to MOET calves. The difference was significant (FDR < 0.1) although, for 10 and 3 of the 12 subunits of EIF3 in liver and muscle, respectively (Table S8).

Therefore, a potential explanation for the observed results is that the “energy” economy mode adopted by the IVP embryos [72], and the subtle but impaired placental perfusion during pregnancy, impacted the liver metabolism during fetal life, increasing glucose production from the liver and leading to increased peripheral insulin sensitivity in postnatal life, in a similar fashion as occurring in IUGR fetuses [73]. Although it was not measured, it is possible that IVP animals presented peripheral insulin resistance, as suggested by the functional analysis of the clusters of NPCG with increased expression in the muscle of IVP calves (Table S7). The metabolic status of these animals could have been modified to prioritize energy production by the TCA cycle and lipid oxidation, rather than protein synthesis, in muscle, whereas both processes were augmented in the liver, of the IVP calves. Furthermore, these IVP animals showed an epigenomic and transcriptomic profile compatible with an earlier activation of the HPG axis [15]. If energy availability is restricted during fetal development, glucose is allocated to the brain, to prioritize the functioning and development of the central nervous system [74]. Thus, inadequate energy utilization during fetal and postnatal life of IVP calves could have impacted brain development and thus, the activation of the HPG axis.

## Conclusion

In this study, we applied multiomics data integration approaches to investigate the muscular and hepatic epigenomic and transcriptomic differences between healthy IVP and MOET male calves. We introduced a novel concept of NPCG resulting from combining differentially observed features after the analyses of both omics. Results from this study show that IVP calves, with similar birth weight and growth rate as MOET calves, presented alterations in the hepatic and muscular epigenome and transcriptome compatible with altered energy regulation, at 3 months of age. Specifically, cellular aerobic respiration is stimulated in the liver and muscle of IVP calves, whereas protein synthesis is increased in the liver but inhibited in the muscle when compared to the MOET animals. Futures studies can help to elucidate the main mechanisms behind these observations and to evaluate the implications of these findings in practical ART based on IVP of cattle embryos.

## Data availability

The data underlying this article are available in the GEO repository and can be accessed with the accession number GSE176219.

## Authors’ contributions

M. B. Rabaglino analyzed the data, interpreted, and visualized the results, and wrote the original article. M. B. Rabaglino and J. Bojsen-Møller Secher collected the samples. P. Hyttel and J. Bojsen-Møller Secher designed the original project. H. N. Kadarmideen designed the study package, supervised the analyses, and interpreted the results. J. Bojsen-Møller Secher, P. Hyttel, and H. N. Kadarmideen improved the original version of the article. All the authors approved the final version.

## Supplementary Material

SuplementalFigureS1_ioac131

SuplementalFigureS2_ioac131

SuplementalFigureS3_ioac131

SuplementalFigureS4_ioac131

SuplementalFigureS5_ioac131

SupTable1_ioac131

SupTable2_ioac131

SupTable3_ioac131

SupTable4_ioac131

SupTable5_ioac131

SupTable6_ioac131

SupTable7_ioac131

SupTable8_ioac131
