# Supplementary figures and images for: In vitro- and in vivo-produced male dairy calves show molecular differences in the hepatic and muscular energy regulation
Source: Biol Reprod. 2022 Jun 29;107(4):1113–24. doi: 10.1093/biolre/ioac131 (PMC9562124; doi:10.1093/biolre/ioac131)

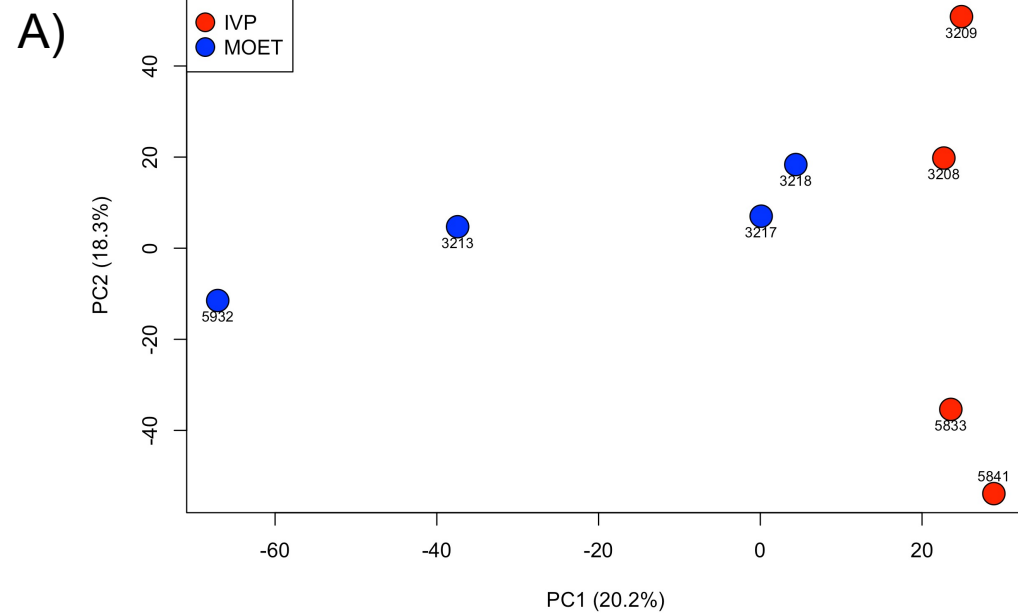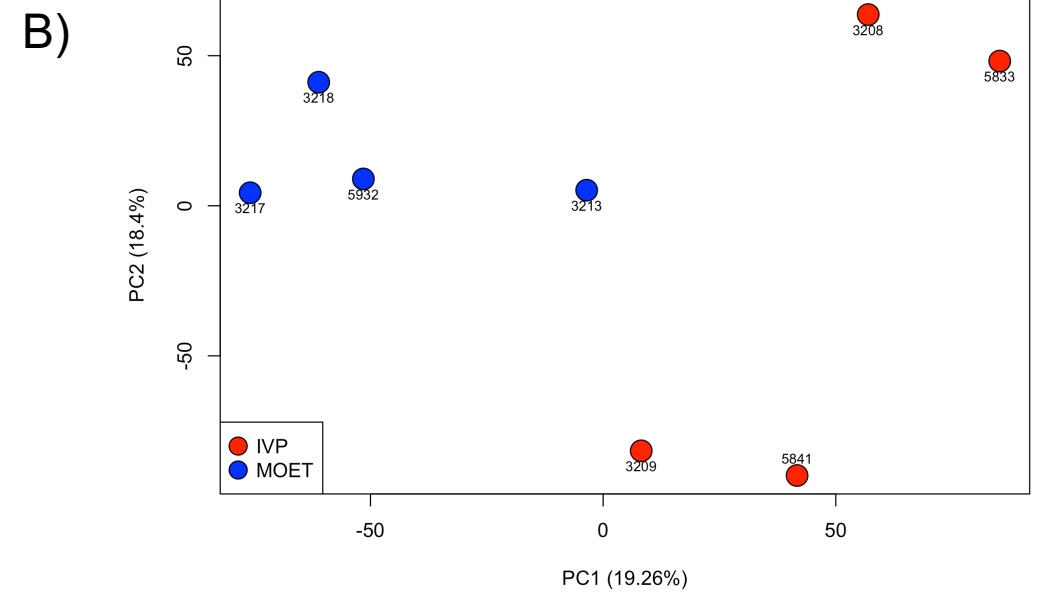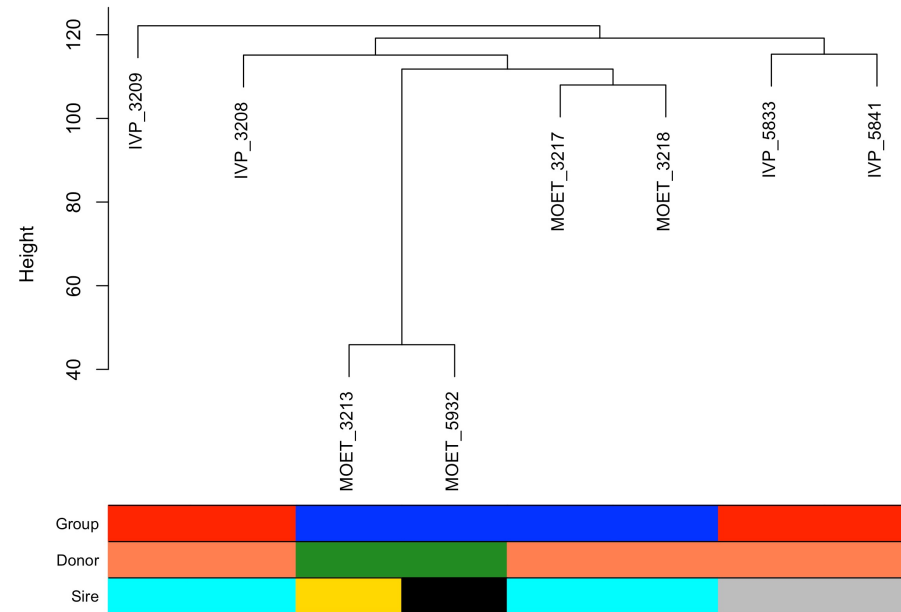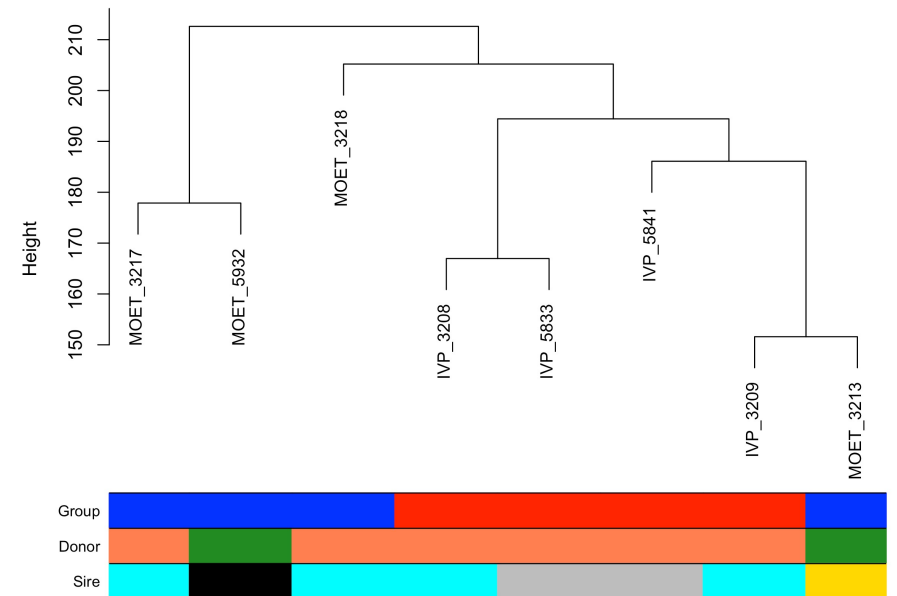

Supplement: SuplementalFigureS1_ioac131 [file suplementalfigures1_ioac131.pdf]

A)

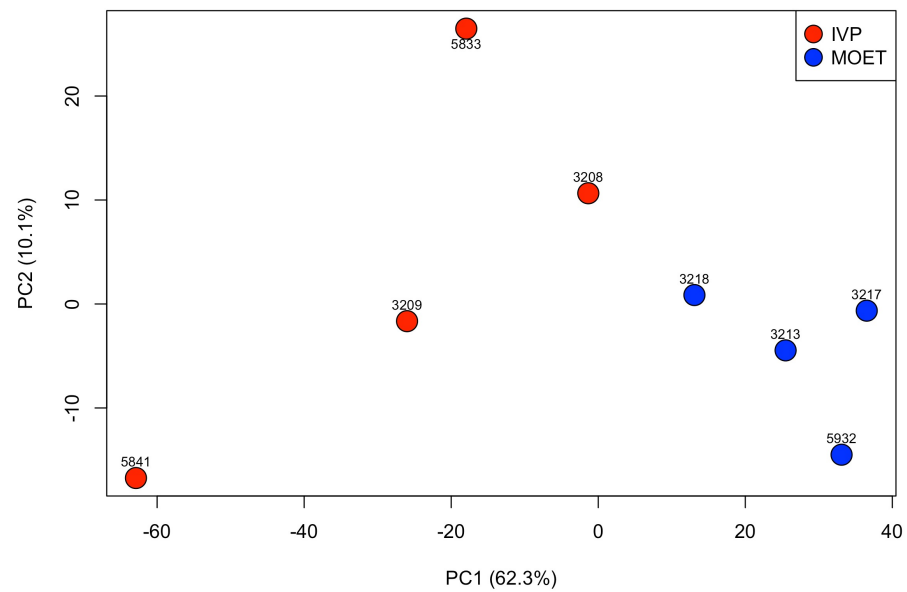

B)

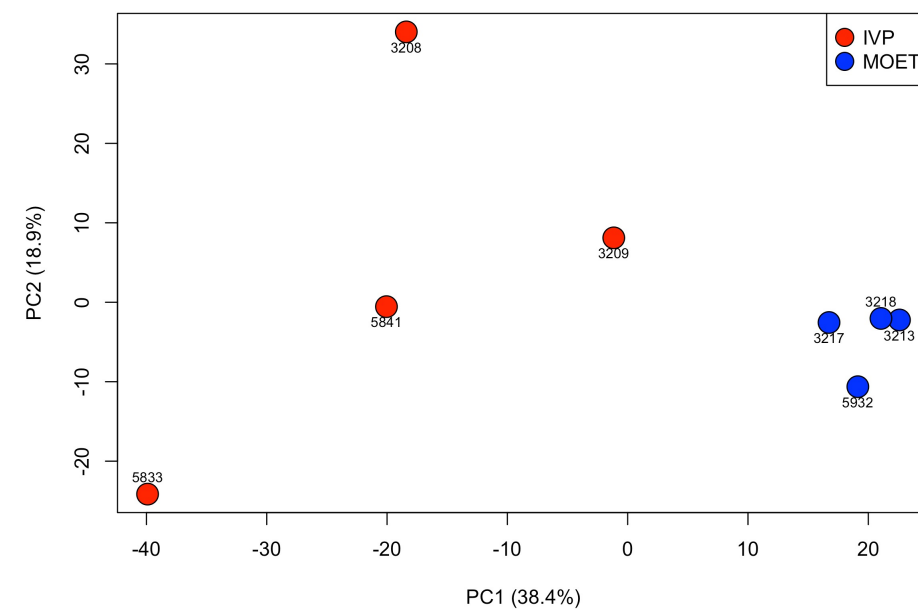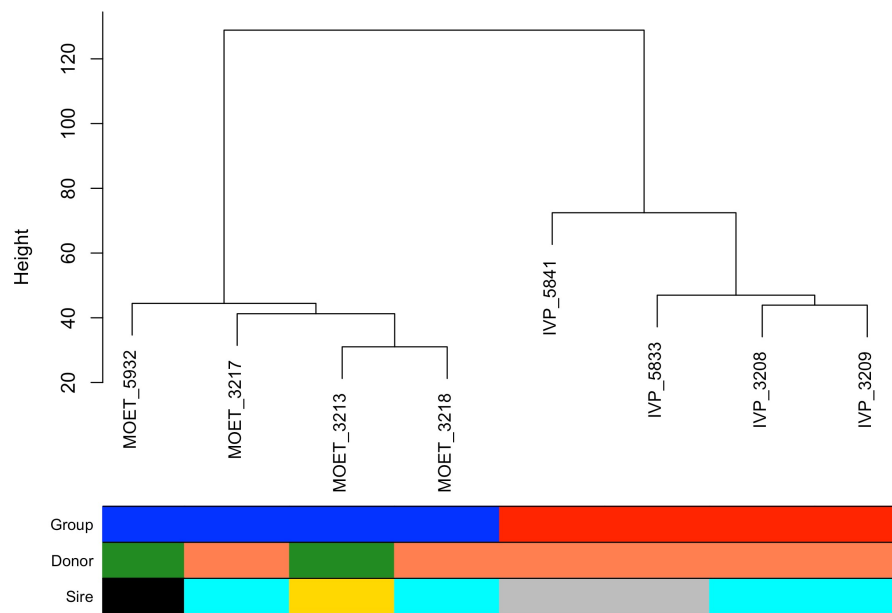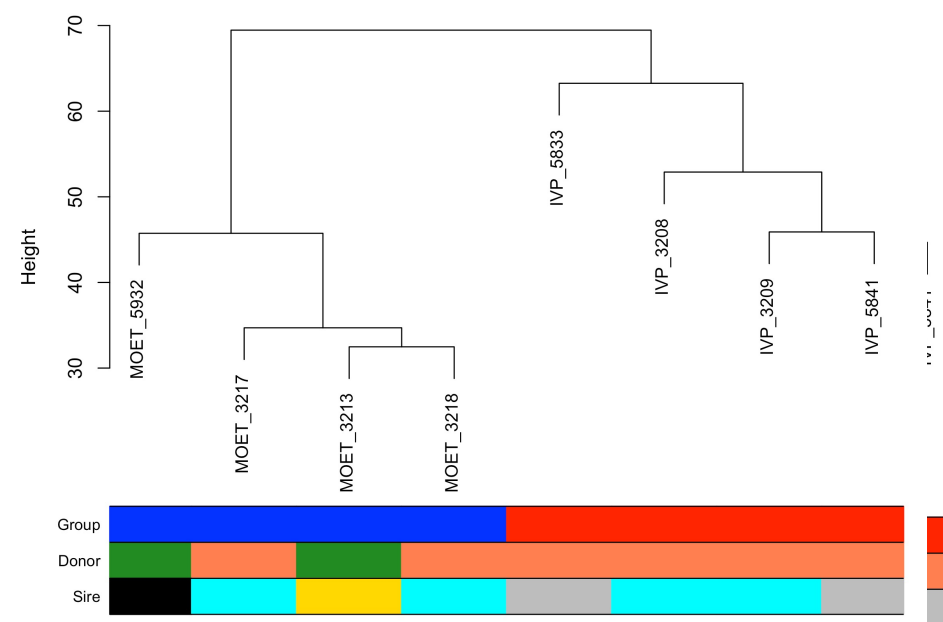

Supplement: SuplementalFigureS2_ioac131 [file suplementalfigures2_ioac131.pdf]

A)

**NPCG**

**OverlapDMC&DEG**

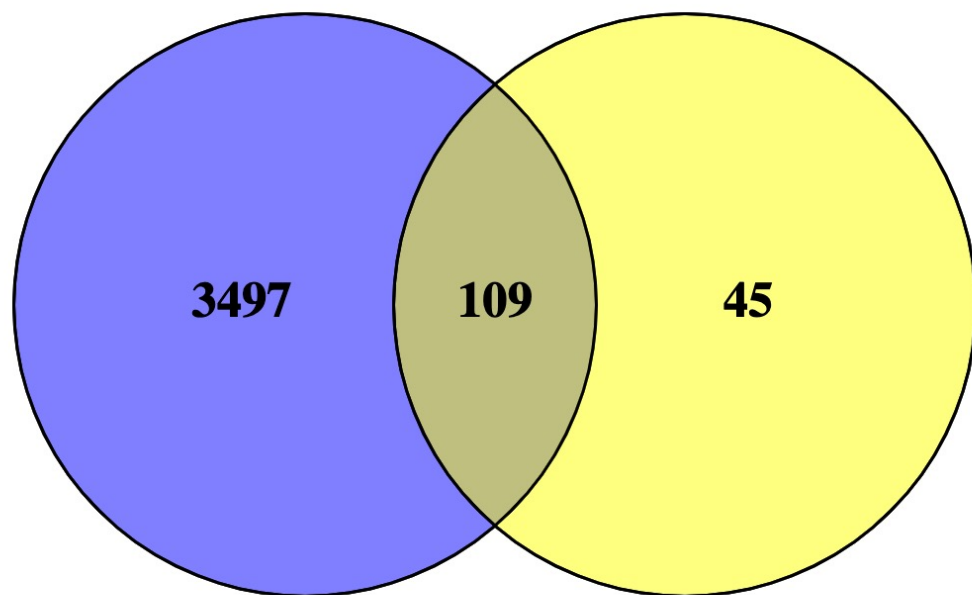

B)

**NPCG**

**OverlapDMC&DEG**

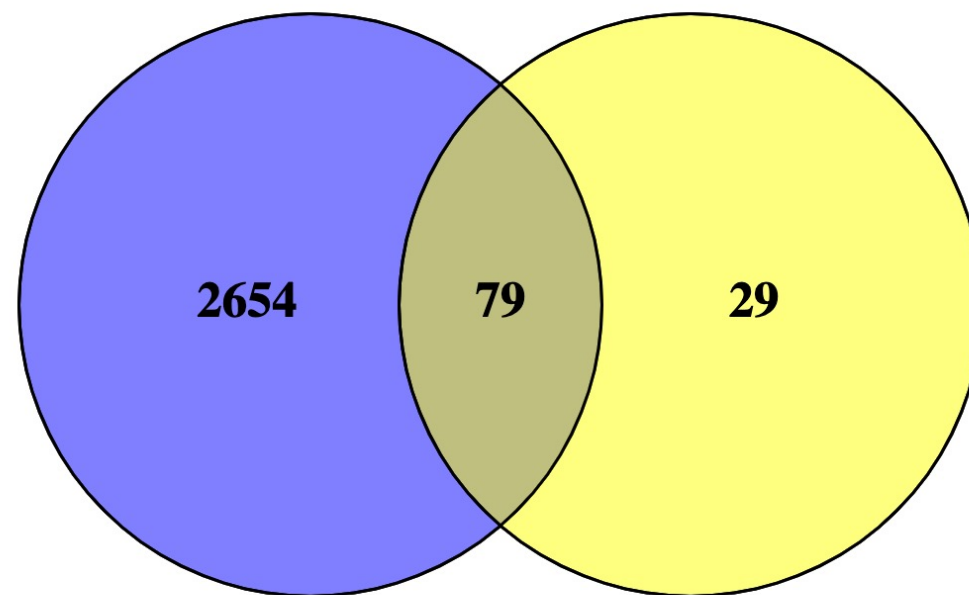

Supplement: SuplementalFigureS3_ioac131 [file suplementalfigures3_ioac131.pdf]
