# Supplementary material for: In vitro- and in vivo-produced male dairy calves show molecular differences in the hepatic and muscular energy regulation
Source: Biol Reprod. 2022 Jun 29;107(4):1113–24. doi: 10.1093/biolre/ioac131 (PMC9562124; doi:10.1093/biolre/ioac131)
Supplement: SuplementalFigureS5_ioac131 [file suplementalfigures5_ioac131.pdf]

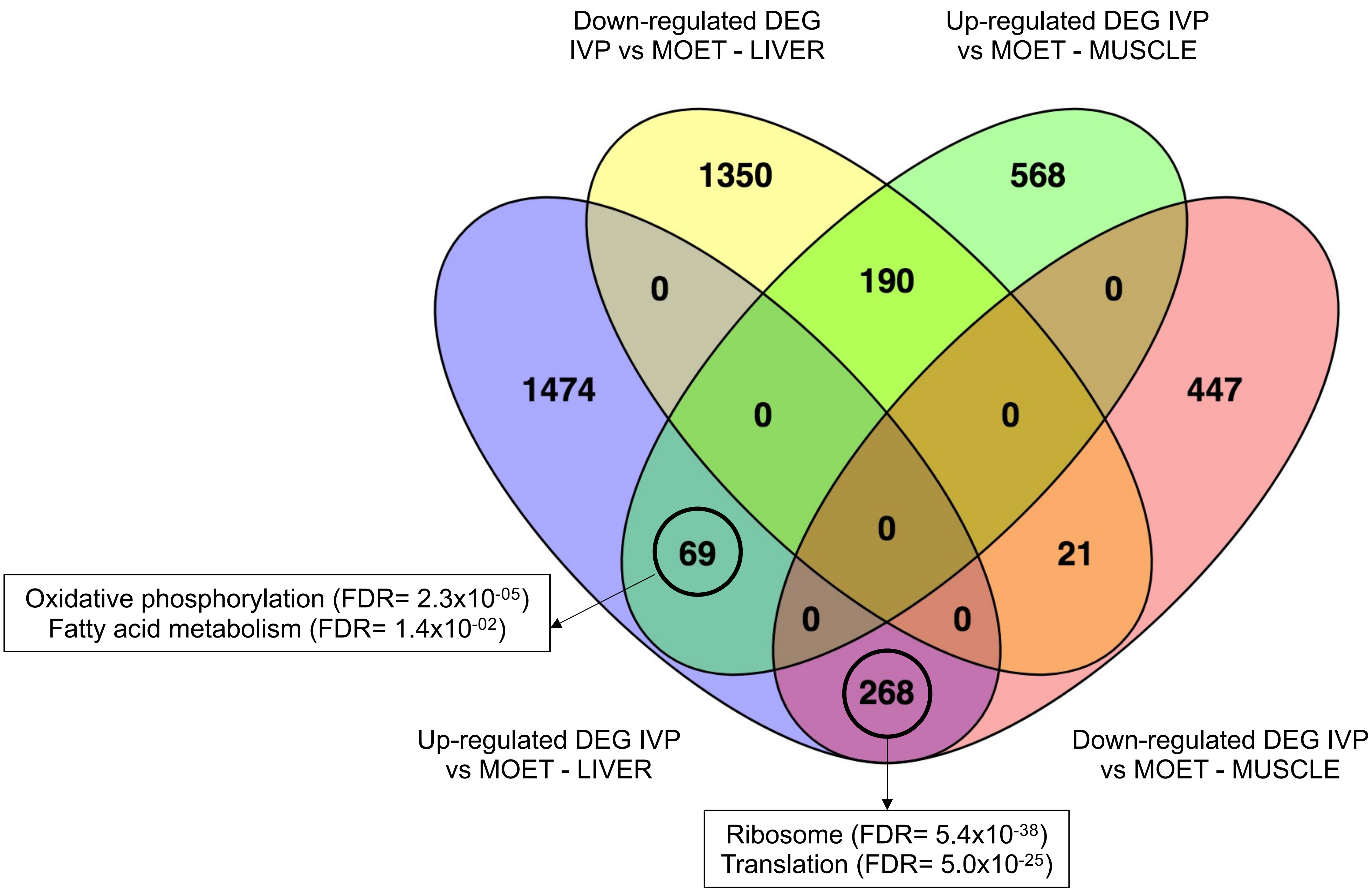
Down-regulated DEG IVP vs MOET - LIVER
Up-regulated DEG IVP vs MOET - MUSCLE
1350
568
0
190
0
1474
0
0
447
0
69
21
0
0
268
Oxidative phosphorylation (FDR= 2.3x10-05)
Fatty acid metabolism (FDR= 1.4x10-02)
Up-regulated DEG IVP vs MOET - LIVER
Down-regulated DEG IVP vs MOET - MUSCLE
Ribosome (FDR= 5.4x10-38)
Translation (FDR= 5.0x10-25)
