# Supplementary material for: In vitro- and in vivo-produced male dairy calves show molecular differences in the hepatic and muscular energy regulation
Source: Biol Reprod. 2022 Jun 29;107(4):1113–24. doi: 10.1093/biolre/ioac131 (PMC9562124; doi:10.1093/biolre/ioac131)
Supplement: SuplementalFigureS4_ioac131 [file suplementalfigures4_ioac131.pdf]

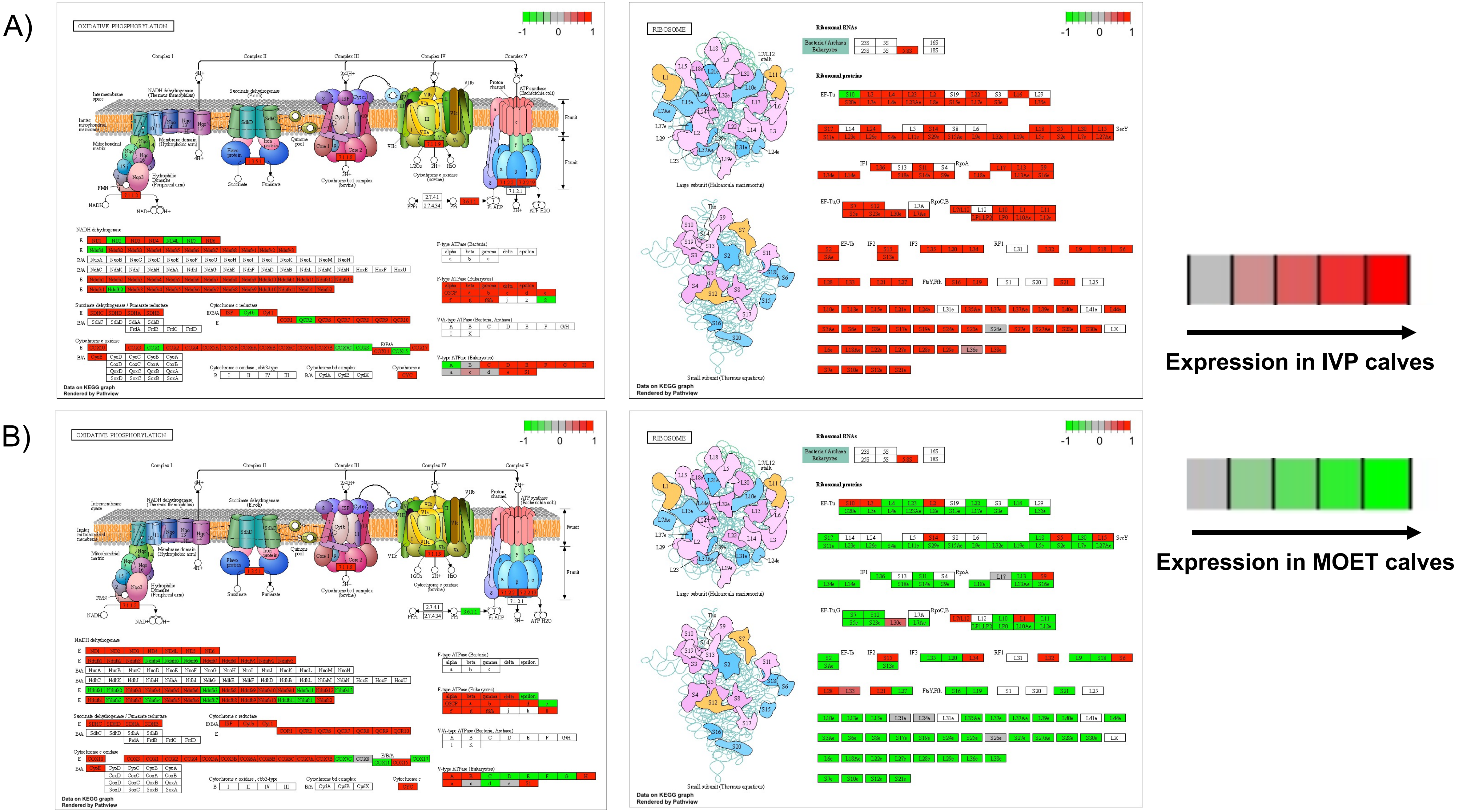
A)
B)
OXIDATIVE PHOSPHORYLATION
RIBOSOME
-1 0 1
Data on KEGG graph
Rendered by Pathview
Expression in IVP calves
Expression in MOET calves
